# The prediction of Chongqing's GDP based on the LASSO method and chaotic whale group algorithm–back propagation neural network–ARIMA model

**DOI:** 10.1038/s41598-023-42258-z

**Published:** 2023-09-11

**Authors:** Juntao Chen, Jibo Wu

**Affiliations:** School of Mathematics and Big Data, Chongqing University of Arts and Sciences, Chongqing, 402160 China

**Keywords:** Applied mathematics, Statistics

## Abstract

Accurate GDP forecasts are vital for strategic decision-making and effective macroeconomic policies. In this study, we propose an innovative approach for Chongqing's GDP prediction, combining the LASSO method with the CWOA—BP–ARIMA model. Through meticulous feature selection based on Pearson correlation and Lasso regression, we identify key economic indicators linked to Chongqing's GDP. These indicators serve as inputs for the optimized CWOA–BP–ARIMA model, demonstrating its superiority over Random Forest, MLP, GA–BP, and CWOA–BP models. The CWOA–BP–ARIMA model achieves a remarkable 95% reduction in MAE and a significant 94.2% reduction in RMSE compared to Random Forest. Furthermore, it shows substantial reductions of 80.6% in MAE and 77.8% in RMSE compared to MLP, along with considerable reductions of 77.3% in MAE and 75% in RMSE compared to GA–BP. Moreover, compared to its own CWOA–BP counterpart, the model attains an impressive 30.7% reduction in MAE and a 20.46% reduction in RMSE. These results underscore the model's predictive accuracy and robustness, establishing it as a reliable tool for economic planning and decision-making. Additionally, our study calculates GDP prediction intervals at different confidence levels, further enhancing forecasting accuracy. The research uncovers a close relationship between GDP and key indicators, providing valuable insights for policy formulation. Based on the predictions, Chongqing's GDP is projected to experience positive growth, reaching 298,880 thousand yuan in 2022, 322,990 thousand yuan in 2023, and 342,730 thousand yuan in 2024. These projections equip decision-makers with essential information to formulate effective policies aligned with economic trends. Overall, our study provides valuable knowledge and tools for strategic decision-making and macroeconomic policy formulation, showcasing the exceptional performance of the CWOA–BP–ARIMA model in GDP prediction.

## Introduction

Gross Domestic Product (GDP) is an indicator that measures the value of all goods and services produced by a country or region during a specific period and is crucial for economic development and strategic planning^1^. Accurately predicting GDP is crucial for understanding economic growth patterns, promoting economic reforms, and making decisions during model transitions^2^. Analyzing historical GDP data helps to determine development trends and formulate relevant policies^3^.

However, the existing GDP forecasting methods mainly rely on a single model, such as Autoregressive Integrated Moving Average (ARIMA)^4^, Backpropagation Neural Network (BPNN)^5^, and grey forecasting^6^, which cannot accurately capture the impact of nonlinear factors. Ignoring forecast errors and relying on a single model may lead to inaccurate GDP forecasts^7^. In order to improve our understanding and prediction of GDP, we need to use scientific methods and historical GDP data to predict future value^8^, providing valuable insights for the public economy^9^. As the fourth largest city in China's provincial-level administrative region, Chongqing plays an important role in the national Western development strategy, and its contribution to the GDP of the Western region continues to increase. Therefore, studying and predicting Chongqing's GDP is significant^10^.

The development of neural networks has seen significant milestones in the field of artificial intelligence. Robert Hecht Nielson proved for the first time that the three-layer neural network could fit any nonlinear continuous function^11^. Since the 1990s, Lapeds and Farber have started applying artificial neural networks to the prediction field^12^. Afterward, Werbos and other researchers validated artificial neural networks' self-learning and nonlinear advantages and extended them to time series prediction in 2012^13^. Li Cong combined backpropagation (BP) and empirical mode decomposition (EMD) algorithms to predict the price of stock index futures^14^. In 2013, Yang Fan used a genetic algorithm to optimize some parameters of the BP neural network and predict the closing price of the stock market^15^. In 2015, Feng Peng combined the particle swarm optimization (PSO) algorithm and BP neural network to predict network traffic using the PSO–BP neural network model^16^. In recent years, researchers have increasingly turned to combining various information sources into composite models to improve the accuracy of GDP forecasting. For example, Qin et al. developed a seasonal error-corrected time prediction model using SARIMA–BP, which effectively combines linear and nonlinear components^17^. You constructed the ARIMA model and utilized a BP neural network to explore the impact of algorithm structure on GDP prediction^18^. The author used a fusion model that fused Chinese consumer price index (CPI) data for prediction^19^. Qing applied the ARIMA–BPNN model to predict CPFR demand^20^.

To address the limitations of current methods and enhance the accuracy of Chongqing's GDP prediction, this study proposes a novel approach that integrates chaotic optimization technology. Drawing inspiration from recent research, this study is influenced by the hybrid estimation model VMD–WOA–LSTM, which accurately estimates monthly evapotranspiration (ET) using deep learning LSTM, variational mode decomposition (VMD), and the whale optimization algorithm (WOA)^21^. Additionally, Yu et al. demonstrated the potential of chaotic optimization in improving prediction models through their work on ultra-short-term wind power prediction. In their study, they proposed a new framework based on RF–WOA–VMD and BiGRU attention mechanism optimization.^22^ and Altan and Karasu (2020) developed a hybrid model combining 2D curvelet transform, chaotic salp swarm algorithm, and deep learning technique for the recognition of COVID-19 disease from X-ray images^23^. Furthermore, we incorporate insights from the study emphasizing the significance of chaotic optimization in enhancing prediction performance^24,25^. By referencing these research findings, we aim to effectively showcase our research's driving force and unique contributions. This study has the following attractive characteristics:

1. This paper introduces the CWOA–BP–ARIMA model for accurate and reliable GDP forecasts, outperforming benchmark models and reducing Mean Absolute Error (MAE).
2. Key economic indicators are identified and integrated using advanced techniques, enhancing the neural network model's input for more precise and robust forecasts.
3. Robust prediction intervals are calculated, providing decision-makers with a comprehensive range of forecasted GDP values for risk assessment and strategic decision-making.
4. Through comparative analysis, the CWOA–BP–ARIMA model's superior performance is validated, offering valuable insights into Chongqing's economic landscape.

## Methodology

### Feature selection method

#### Pearson correlation coefficient

The linear correlation between two variables can be calculated and measured using the traditional PCC approach. PCC has a value between − 1 and 1; the more significant the absolute value, the stronger the correlation. The PCC between X and Y is shown in Eq. (1).

1 $$ \rho_{XY} = \frac{{E\left[ {\left( {X - \mu_{X} } \right)\left( {Y - \mu_{Y} } \right)} \right]}}{{\sqrt {D_{X} D_{Y} } }} $$

where represents the covariance of X and Y, the standard deviation of X and Y, the mean of X and Y; and the variances of X and Y, respectively.

#### Lasso method

In the era of rapid development of information technology, it is easier to obtain data. However, in modeling specific problems, we may encounter computational complexity, high-dimensional data is challenging to deal with, and so on. To consider the factors that affect the research variables as much as possible, we often add more independent variables to achieve a more accurate prediction model. However, too many independent variables will lead to variable redundancy, and not all data are related to the research object. Therefore, the choice of variables is critical in studying a specific issue.

In response to this problem, Robert Tibshirani proposed the Lasso method in 1996, summarized explicitly as imposing a penalty on the model coefficients' absolute value function for compressing the model coefficients enabling variable selection. The model structure is as follows:

2 $$ \hat{\beta } = \arg \min \left\{ {\sum\limits_{t = 1}^{N} {\left( {y_{i} - \sum\limits_{j = 1}^{p} {\beta_{j} x_{ij} } } \right)^{2} + \lambda \sum\limits_{j = 1}^{p} {\left| {\beta_{j} } \right|} } } \right\} $$

### Basic model overview

#### BP neural network

**Figure 1:**
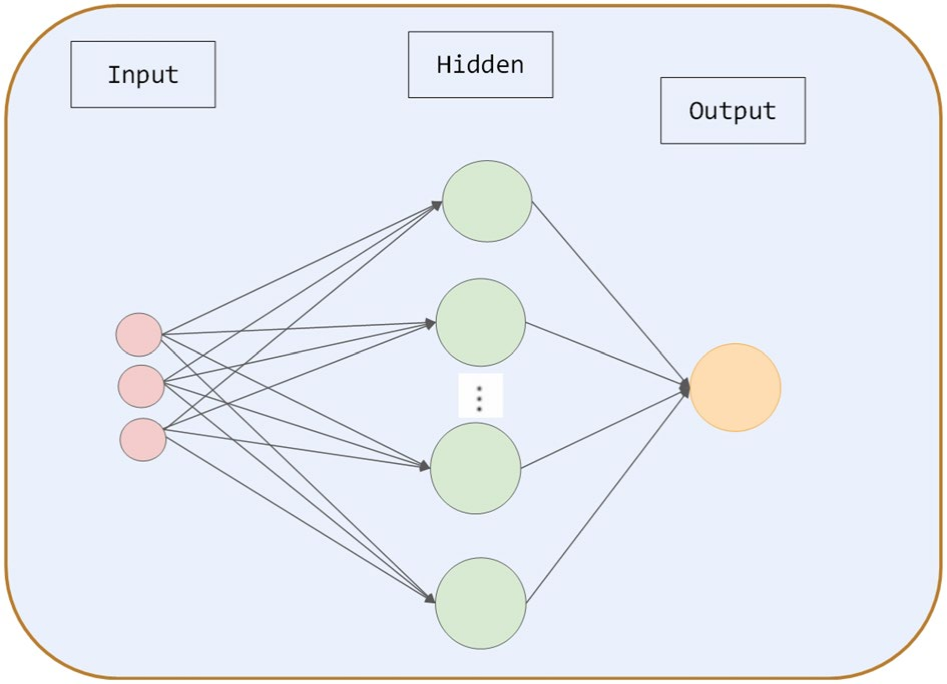
BPNN structure.

The ability of an ANN to mimic the actions of an information system called an animal neural network is one of its traits. Artificial neural networks were conceptualized as systems that could do complicated tasks by replicating the nervous system of human brains. ANN is effective at solving nonlinear issues. As far as we know, ANN can produce dozens of representative models, the most popular being BPNN and its extended forms. Figure 1 shows the structure of the BPNN used in our proposed model. Through "training" events, the BP algorithm determines whether the relationship between the input and output should be linear or nonlinear. There are two phases to the "training" procedure: forward and reverse propagation^26^.

Backward propagation phase—error propagation phase:

1. Calculate the difference between the actual output $Q_{p}$ and the output $O_{i}$;
2. Adjusting output layer weight matrix by output layer error;
3. $$E_{i} = \frac{1}{2}\sum\limits_{j = 1}^{n} {\left( {Q_{ij} - O_{ij} } \right)^{2} }$$
4. To estimate the error of the preceding layer, we use the error of the direct leading layer of the output layer, and to estimate the error of the direct leading layer, we use the error of the leading layer of the output layer.
5. By adjusting the weight matrix based on these estimations, the error at the output end is gradually propagated back toward the source.

#### ARIMA model

ARIMA, a popular model for time series analysis and prediction^27^, has a significant advantage when dealing with linear time series. This method makes the non-stationary time series a stationary time series. The model combines two models by regressing the lag value of the dependent variable and its random error: Moving Average and Autoregressive Random variable dependencies between groups include internal and external influencing factors. This method helps to explain the law of prediction changes and has a high prediction accuracy. The best option is to guarantee that while estimating, the ARIMA model's time series is fixed or around fixed, and the autocorrelation coefficient has just a single variable, the time stretch, or at least, the time change doesn't influence the mean and difference.

The ARIMA model, also known as the autoregressive integrated moving average model, considers the difference $\Delta X_{t} = X_{t} - X_{t - 1} = \left( {1 - L} \right)X_{t}$, when the time series itself is not stationary. $\left\{ {X_{t} } \right\}$ can be viewed as a stationary sequence.

The ARIMA (p, d, q) model is expressed as:

3 $$ W_{t} = \varphi_{1} W_{t - 1} + \varphi_{2} W_{t - 2} + \varphi_{3} W_{t - 3} + \cdots \varphi_{p} W_{t - p} + \theta_{1} \varepsilon_{t - 1} + \cdots \theta_{q} \varepsilon_{t - q} $$

#### Error variance mean square reciprocal

In this paper, the error variance means square reciprocal method combined forecasting model will combine the single CWOA–BP and ARIMA models and give them the corresponding weights so that the forecasting results are more accurate. The calculation formula is as follows:

4 $$ \begin{gathered} W_{j} = \frac{{E_{j}^{ - 1/2} }}{{\sum\limits_{j = 1}^{J} {E_{j}^{ - 1/2} } }},j = 1,2,3,...,J \hfill \\ S_{i} = \sqrt {\frac{1}{m - 1}\sum\limits_{i = 1}^{m} {\left( {y_{i} - \overline{y}} \right)^{2} } } ,i = 1,2,3,...,m \hfill \\ \end{gathered} $$

where represents the error variance of the J-th prediction model, represents the weight, and the expression is:

5 $$ \hat{Y} = \sum\limits_{i}^{n} {W_{i} } Y_{i} ,i = 1,2,3,...,n $$

#### Whale optimization algorithm

**Figure 2:**
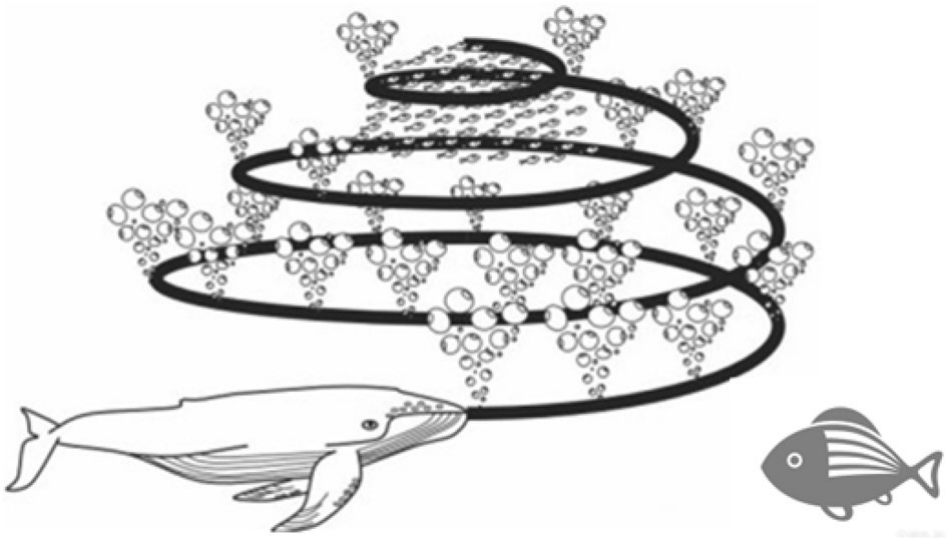
Humpback comparison of Bubble-net foraging behavior^28^.

Mirjalili et al.^28^ proposed the whale optimization approach based on the whale swarm intelligent optimization algorithm. The whales are widely recognized as the largest mammals on Earth and possess a remarkable ability to employ echolocation for prey detection and communication among themselves. Among the whale species, killer whales and other toothed cetaceans have developed impressive hunting skills. On the other hand, baleen whales, such as the humpback whale, lack teeth and have adapted to prey on small schools of fish and shrimp. To facilitate this feeding process, they have evolved a unique foraging behavior known as bubble-net foraging, also referred to as bubble-net foraging or air curtain foraging, as illustrated in Fig. 2.

WOA algorithm abstraction out of 3 behaviors: entrapment prey, bubble-net attack, and random search.

##### Enclosure of prey

Whales are able to identify prey location and surround prey through echolocation, and the equation expression for encircling prey behavior is:

6 $$ W\left( {t + 1} \right) = W^{*} \left( t \right) - AD $$

7 $$ D = \left| {CW^{*} \left( t \right) - W\left( t \right)} \right| $$

where t is the current iteration, and $W^{*} \left( t \right)$ denotes the current optimal. The coefficient vectors A and C are defined as follows:

8 $$ A = 2a{\text{rand}}_{1} - a $$

9 $$ C = 2{\text{rand}}_{2} $$

10 $$ a = 2 - {{2t} \mathord{\left/ {\vphantom {{2t} {t_{\max } }}} \right. \kern-0pt} {t_{\max } }} $$

where: ${\text{rand}}_{1}$ and ${\text{rand}}_{2}$ are the uniform-sentence distributions in the range [0,1].

random numbers: a is the convergence factor. It decreases linearly from 2 to 0 with the number of iterations 0, $t_{\max }$ is the maximum number of iterations.

##### Bubble-net attacks

In order to describe mathematically Bubble-net foraging behavior, design contraction enveloping mechanism and spiral update Location two methods. In the spiral update position method, the whale moves in a spiral motion towards its prey swimming, whose mathematical model is:

11 $$ X^{t + 1} = X_{gbest}^{t} + De^{bt} \cos \left( {2\pi l} \right) $$

In this formula,$D = \left| {X_{gbest}^{t} - X^{t} } \right|$ is the whale and the current global optimal individual. The distance between b is the constant that defines the shape of the logarithmic spiral and l is a random number between [− 1,1].

##### Searching for prey

Humpback whales exhibit random swimming patterns that are influenced by their positions relative to one another. This behavior can be mathematically represented by the following model when they are searching for prey:

12 $$ D = \left| {CW_{rand} \left( t \right) - W\left( t \right)} \right| $$

13 $$ W\left( {t + 1} \right) = W_{rand} \left( t \right) - AD $$

This formula $W_{rand} \left( t \right)$ represents the randomly chosen whale positions. When |A|> 1, the whales are forced to stay away from their prey and update all whale positions with the randomly generated ones $W_{rand} \left( t \right)$.

#### Chaotic whale optimization algorithm (CWOA)

The WOA algorithm is distinguished by its simple theory, minimum setting of parameters, and focus on dependable performance. WOA has a reasonable convergence rate but needs help finding a globally optimal solution that might further increase the algorithm's convergence speed. To counteract this impact and improve the WOA algorithm's performance, the CWOA algorithm was developed by introducing chaos into the original algorithm. In computer science, the term "map" describes associating a function with a particular element in a chaotic algorithm. With its ergodicity and non-repetition properties, chaos may execute comprehensive searches faster than stochastic searches, which rely exclusively on probability.

**Figure 3:**
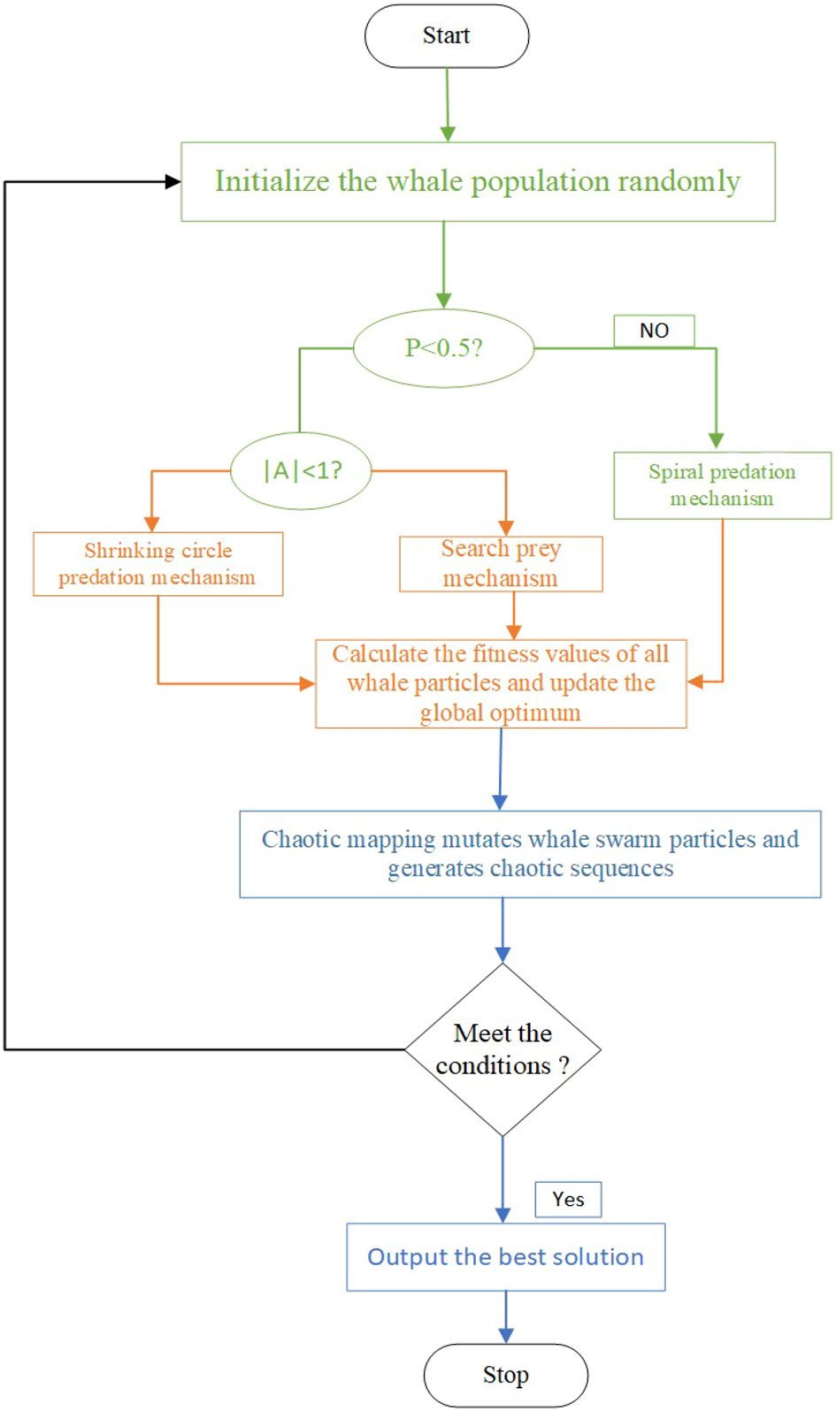
Flow chart of Chaotic Whale Optimization Algorithm.

The quality of the initial population is crucial for the accuracy and convergence speed of the algorithm^29^. In the case of the WOA algorithm, randomly generated initial populations can lead to limited diversity and uneven distribution. The Chaotic Whale Optimization Algorithm (CWOA) incorporates a chaotic reverse learning initialization strategy to address this. By leveraging the random, exploratory, and regular characteristics of chaotic variables, the CWOA generates an initial population with improved diversity. This is achieved by selecting solutions with higher fitness values from the chaotic initial and reverse populations. The CWOA's initialization process, depicted in Fig. 3, ensures an optimized initial population, enhancing the algorithm's comprehensive global search ability. With the CWOA's initialization strategy, the algorithm benefits from enhanced population diversity and improved efficiency in solving optimization problems, the initial parameters for these two algorithms are shown in Table 1. The Tent chaotic mapping function expression^30^ is:

14 $$ y_{i + 1,d} = \left\{ {\begin{array}{*{20}l} {2y_{id} ,y_{id} < 0.5} \hfill \\ {2(1 - y_{id} ),y_{id} \ge 0.5} \hfill \\ \end{array} } \right. $$

**Table 1:** Explanatory variables.

Variable name	Meaning of variable
X_1_	Registered urban unemployment rate
X_2_	Freight volume (Million tons)
X_3_	Fiscal revenue (CNY ten thousand)
X_4_	Fiscal Expenditure (CNY ten thousand)
X_5_	Total output value of agriculture, forestry, animal husbandry and fishery (CNY ten thousand)
X_6_	Total output value of construction industry (CNY ten thousand)
X_7_	Total retail sales of social consumer goods (CNY ten thousand)
X_8_	Total import and export value (CNY ten thousand)

The chaotic sequence is mapped into the solution space to obtain a population.

$X = \left\{ {X_{i} ,i = 1,2,...,N} \right\},X_{i} = \left\{ {X_{id} ,d = 1,2,...,D} \right\}$, and the population individual $X_{id}$ is expressed as:

15 $$ X_{id} = X_{\min d} + y_{id} \left( {X_{\max d} - X_{\min d} } \right) $$

$X_{id}$ is the d-dimensional code value of the i-th population, $X_{\min d}$ and $X_{\max d}$ are the upper and lower bounds of $X_{id}$.

Calculating inverse population $PX = \left\{ {PX_{i} ,i = 1,2,...,N} \right\}$ from population X,$PX_{i} = \left\{ {PX_{d} ,d = 1,2,...,N} \right\}$,the reverse population individual $PX_{id}$ is denoted by

16 $$ PX_{id} = X_{\min d} + X_{\max d} - X_{id} $$

### The frame of this paper

**Figure 4:**
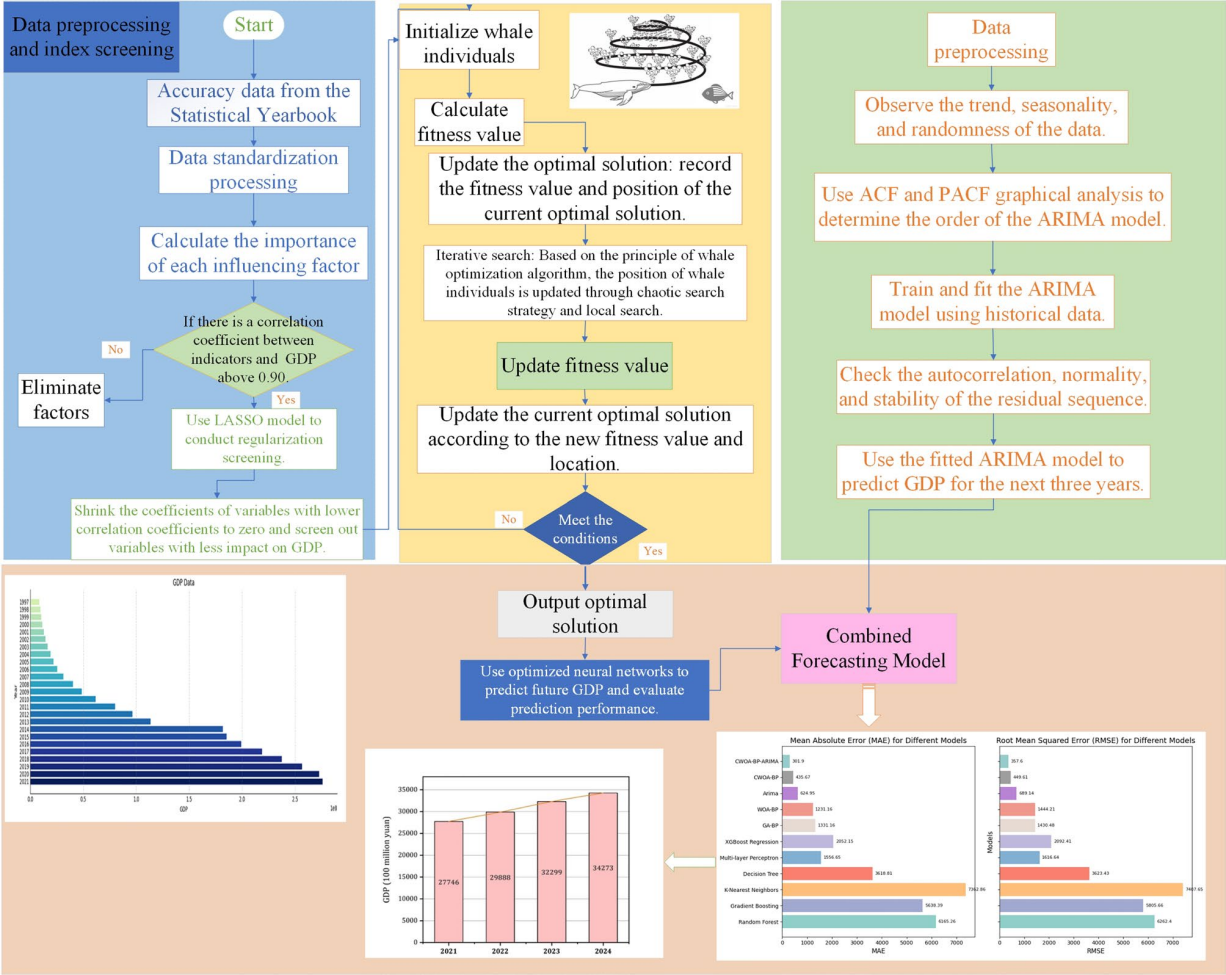
The overall framework of the proposed model for point and interval GDP forecast.

In this paper, the overall framework of the proposed model for point and interval GDP forecast is shown in Fig. 4.

1. The framework starts by collecting economic indicators related to GDP from the Statistical Yearbook. These indicators are then normalized for consistency. Using the Pearson correlation coefficient and LASSO regularization, the most influential factors impacting GDP are identified, ensuring a focused feature selection.
2. The selected features serve as inputs for the CWOA–BP neural network model, a unique combination of the Whale Optimization Algorithm (WOA) and Backpropagation (BP) technique. WOA optimizes the neural network's weights and biases, enhancing performance, while BP captures complex non-linear relationships between features and GDP, making it a powerful tool for prediction.
3. The selected features serve as inputs for the CWOA–BP neural network model, a unique combination of the Whale Optimization Algorithm (WOA) and Backpropagation (BP) technique. WOA optimizes the neural network's weights and biases, enhancing performance, while BP captures complex non-linear relationships between features and GDP, making it a powerful tool for prediction.
4. The CWOA–BP neural network and ARIMA models are integrated into the CWOA–BP–ARIMA composite model, leveraging both methodologies' strengths to generate more robust and accurate GDP predictions.
5. The proposed CWOA–BP–ARIMA composite model is compared with commonly used GDP forecasting models as baselines, validating its superiority and providing valuable insights into Chongqing's economic landscape.

## Results and discussion

### Data description and preprocessing

**Figure 5:**
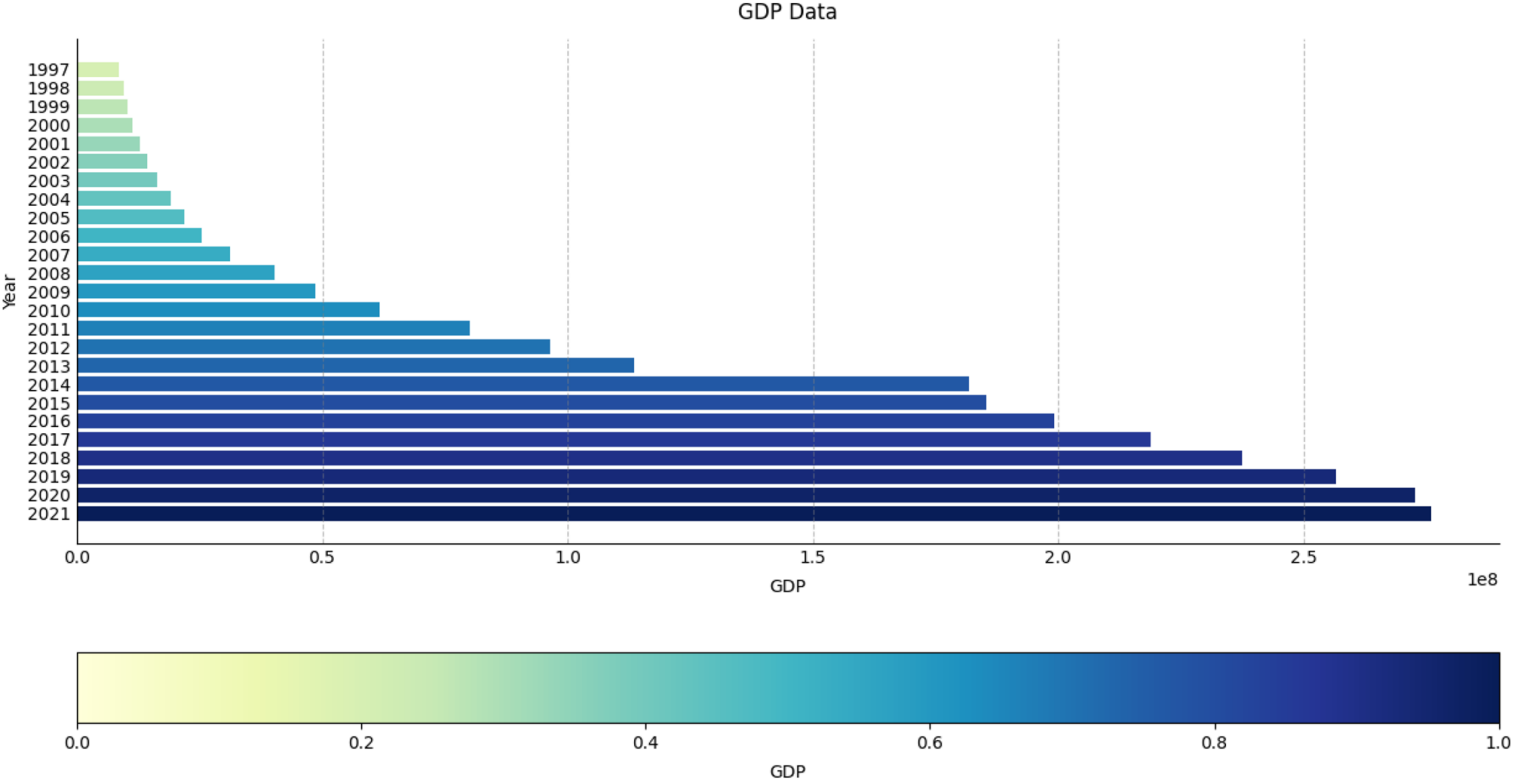
The GDP growth situation of Chongqing from 1997 to 2021.

The study proposes a point and interval GDP forecast model and uses the growth of Chongqing's economy over time as an example to test its dependability and accuracy. First, GDP figures for Chongqing between 1990 and 2021^31^ serve as the research sample; the GDP growth situation from 1997 to 2021 is shown in Fig. 5. In addition, data on GDP from 1990 to 2018 is used for training purposes, whereas data for 2019 and 2021 is used for actual testing. Several factors are considered while assessing the outcomes of the model's tests. Finally, the trained model is applied to forecast Chongqing's GDP from 2022 through 2024.

Select an appropriate ARIMA model first. Second, the BP neural network is optimized using a chaotic search technique. To further rectify the error and retrieve all of the sequence information, a neural network is constructed utilizing the test results and the error of neural network fitting.

The GDP data is first adjusted between years to reduce the time needed for the network to converge. Because low learning efficiency is inevitable if all data is normalized to the positive, its primary purpose is to transform the data into a uniform unit of samples. For this reason, we perform normalization on the numerical data. A normalizing formula looks like this:

17 $$ Y_{i} = \frac{{x_{i} - x_{\min } }}{{x_{\max } - x_{\min } }},i = 1,2,3,...,n $$

### Feature selection results based on PCC–LASSO

**Figure 6:**
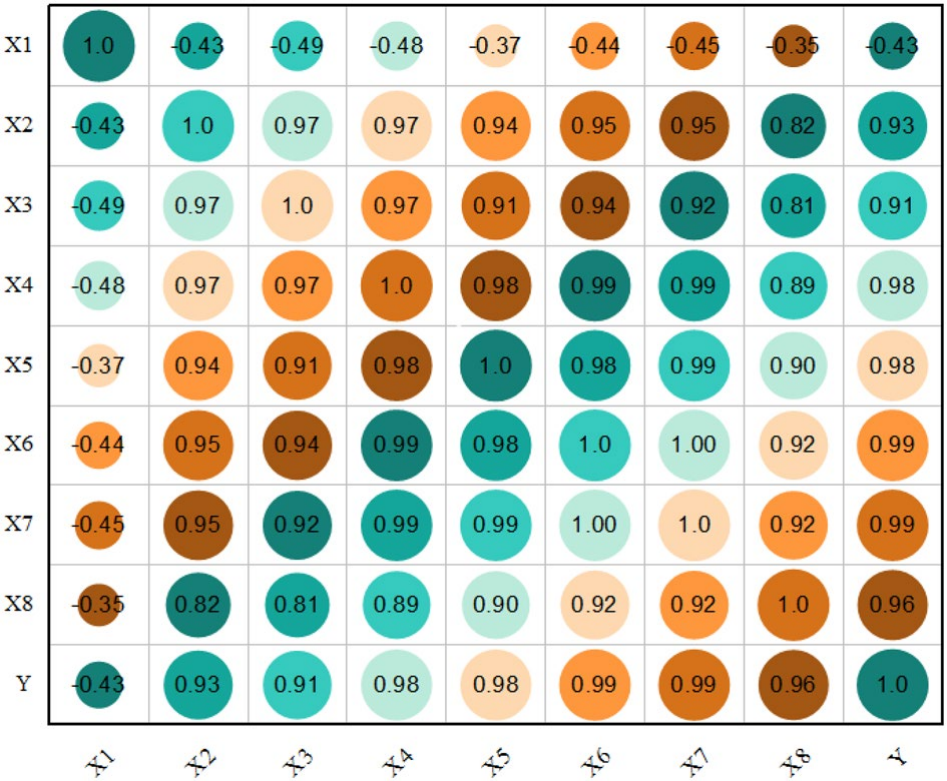
The visual correlation coefficient between GDP and related economic indicators.

This study utilized PCC and LASSO methods to simplify the model to analyze the factors affecting Chongqing's GDP. The results of the PCC method, depicted in Fig. 6, showed that, except for the X_1_ index, all other indicators had correlation coefficients above 0.90 with the economy of Chongqing, indicating a significant linear positive correlation. However, using these indicators as time series variables may result in collinearity and over-fitting issues, which could significantly impact the accuracy of the model's predictions.

To mitigate these concerns, researchers commonly employ regularization techniques like LASSO. This method adds a penalty term to the regression equation, which forces some coefficients to shrink to zero. As a result, the model becomes more straightforward, the number of predictor variables is reduced, and the risks of collinearity and over-fitting are minimized. Therefore, using PCC and LASSO methods can enable researchers to comprehend better the relationship between explanatory variables and Chongqing's GDP while avoiding potential modeling issues. Table 1 provides detailed information on the explanatory variables.

**Figure 7:**
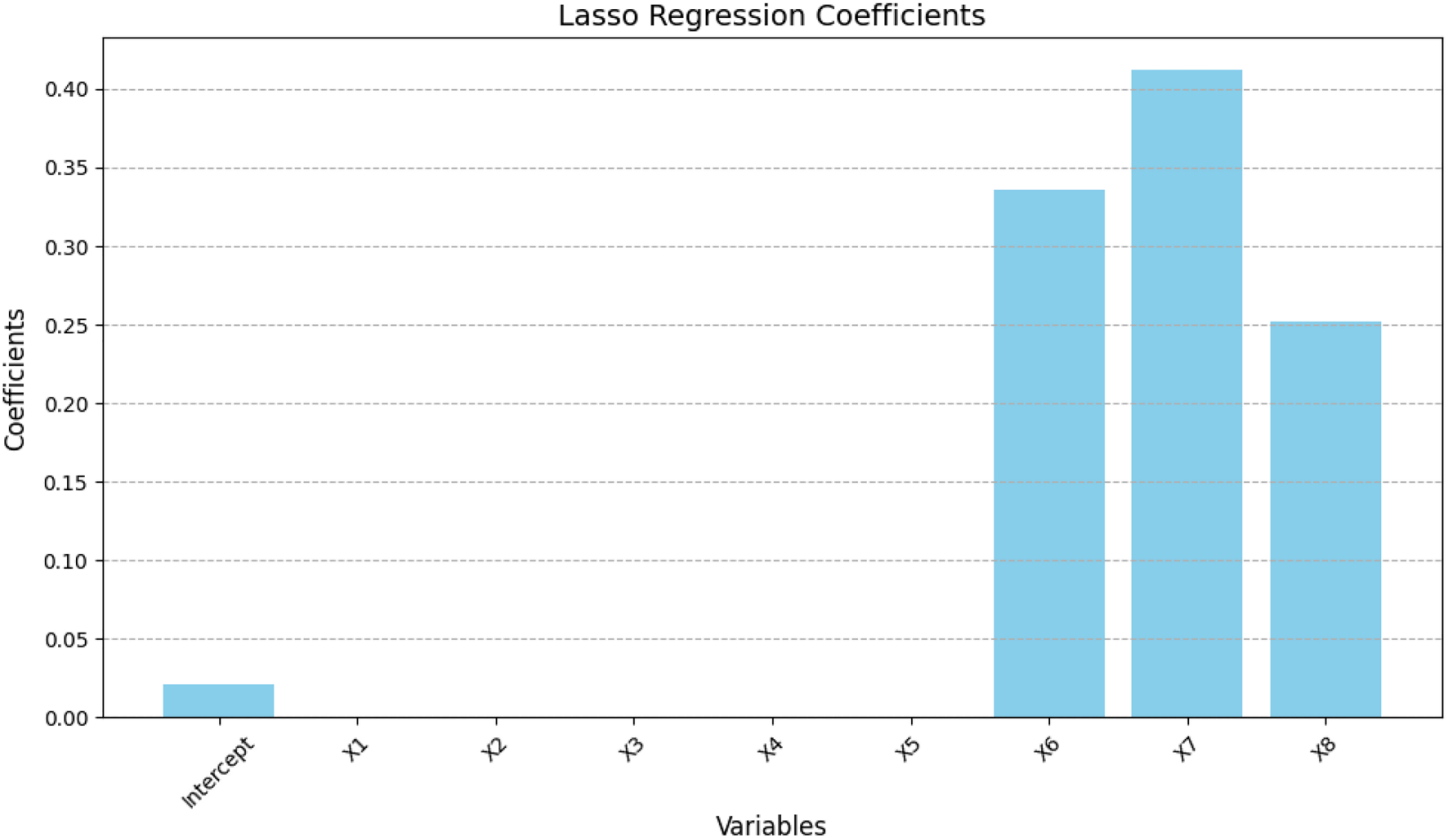
Graph of Lasso Regression Coefficients for Each Variable.

Before re-establishing the model, if the variable selection is performed based on the characteristic of setting the regression coefficient to 0, it can effectively reduce variance, prevent overfitting, solve collinearity problems, and improve prediction accuracy. Through appropriate steps, it was found that with increasing penalties, the regression coefficient of each variable continuously decreases, and the regression coefficient of the final variable is obtained. Figure 7 shows the results of the Lasso variable selection.

### GDP forecasting process based on combined model

#### Empirical analysis of ARIMA model

This study utilized R software to develop exponential smoothing and ARIMA models and used the Box-Jenkins model identification method to determine the best-fit models. Fundamental concepts of this method included examining autocorrelation and partial correlation function diagrams, intuitively understanding sequence truncation and tailing, and selecting five appropriate model types from the sequence. It was essential to evaluate sequence stability before applying the dynamic regression model to avoid breakpoint regression.

Next, further, determine the model parameters. At this point, select the ARIMA (p, d, q) model for forecasting. Manual judgment needs to be more accurate. The parameters are selected from low order to high order according to 0, 1, 2, and 3, and the optimal model is selected according to the AIC criterion.

**Figure 8:**
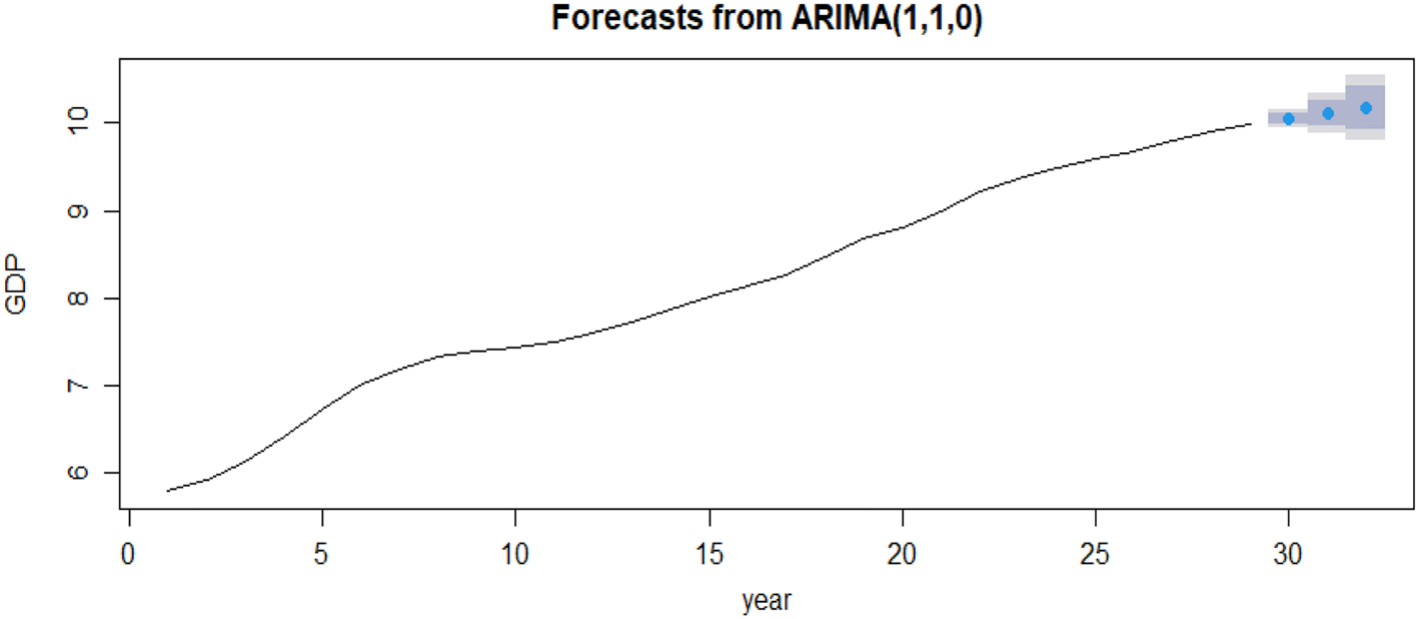
Chongqing GDP forecast 2019–2021.

After the model is established, the model is tested to determine whether the model is significant. They were mainly based on whether the residual sequence of the model is a white noise sequence. After the above tests, using the constructed model to forecast and analyze the test set data for 2019–2021, the GDP forecast table is obtained, as shown in Fig. 8.

Through the establishment of ARIMA, the GDP of Chongqing is predicted. According to Tables 2 and 3, the predicted results are as follows: the relative error of GDP in the 3 years is within 5%. The average absolute percentage error (MAPE = 0.02392), root mean square error (RMSE = 689.141), average absolute error (MAE = 624.95), and coefficient of determination of the economic data sequence are 0.852. The prediction results are good and can be used as the GDP prediction model of Chongqing.

**Table 2:** 2019–2021 GDP forecast value (Unit: 100 million yuan).

Year	Actual value	Predicted value	Error	Relative error (%)
2019	23,605.77	23,117.38	− 488.39	2.06
2020	25,002.79	24,645.02	− 357.77	1.43
2021	27,894.02	26,865.32	− 1028.7	3.68

**Table 3:** 2019–2021 GDP forecast under 80% and 95% confidence intervals (Unit: 100 million yuan).

Year	Actual value	Predicted value	80%Confidence interval	95%Confidence interval
2019	23,605.77	23,117.38	(21,616.86, 24,722.03)	(20,862.38,25,615.94)
2020	25,002.79	24,645.02	(21,292.94, 28,524.82)	(19,707.19, 30,820.03)
2021	27,894.02	26,865.32	(20,610.44, 33,217.11)	(18,164.58, 37,689.62)

#### Empirical analysis of improved CWOA–BP model

The chaotic whale swarm algorithm (CWOA) is a kind of whale swarm algorithm (WOA) based on introducing chaotic mapping to mutate particles. The algorithm is obtained by operating and generating chaotic sequences—the whale swarm algorithm. WOA simulated three predation mechanisms of humpback whales, including searching for predation.

To analyze the impact of different parameters, we employed cross-validation as a robust approach for parameter selection. *Cross-validation* is a statistical technique that helps assess and select the optimal parameter values for a model. The initial parameters are shown in Table 4.

**Table 4:** Initial parameters of each algorithm.

Algorithm	Parameters	Value
WOA	Random value r_1_	[0,1]
	Random value r_2_	[0,1]
	Convergence factor $\alpha$	[0,2]
	Convergence factor b	1
	Random value l	[− 1,1]
CWOA	Random value r_1_	[0,1]
	Random value r_2_	[0,1]
	Convergence factor $\alpha$	[0,2]
	Convergence factor b	1
	Random value l	[− 1,1]

**Figure 9:**
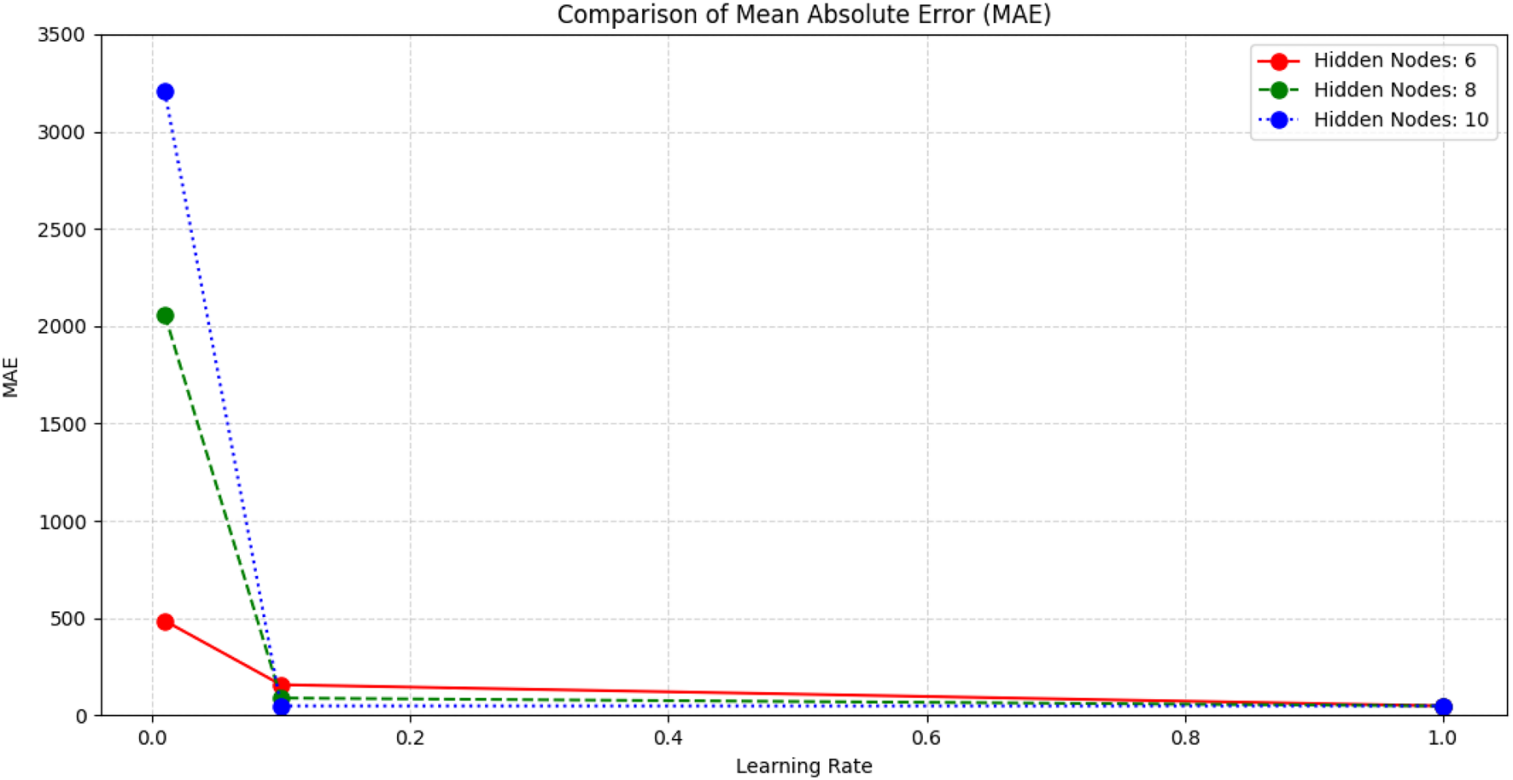
Comparison of MAE results with different hidden nodes and Learning rate.

Our research divided the available data into multiple subsets or folds. Then use, each fold as the validation set, while the remaining folds are used to train the model. By repeating this process and rotating the validation set, we evaluated the model performance more comprehensively under different parameter values. For each parameter setting, we conducted cross-validation and evaluated the model's performance using appropriate indicators such as Mean absolute error (MAE) and mean squared error (MSE). Then, we compared the results obtained from different parameter settings to determine the combination that produces the best performance. Figure 9 visually compares the MAE values of the proposed model under different parameter settings, which helps to select the optimal configuration to improve prediction accuracy.

**Figure 10:**
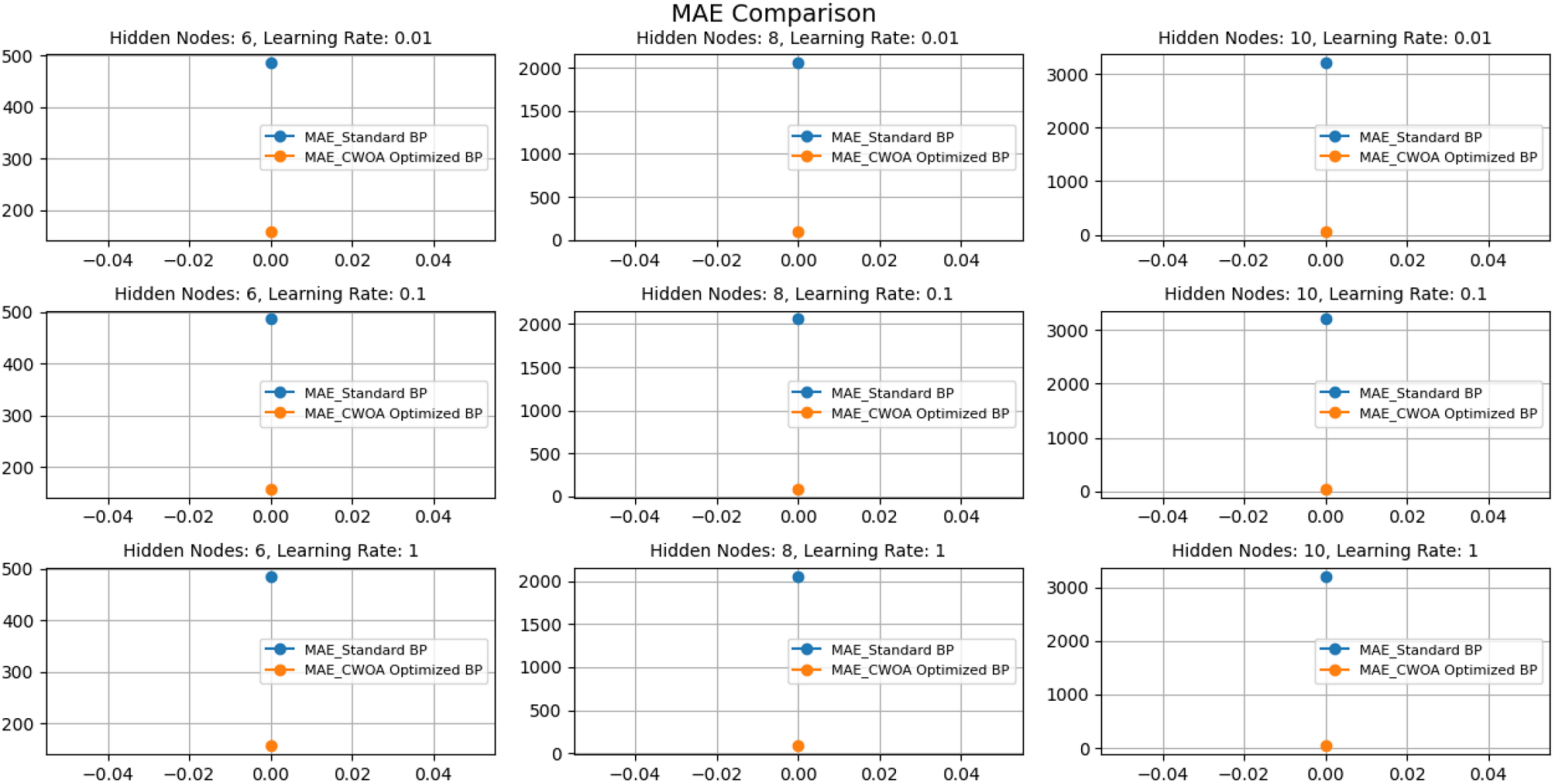
Comparison of MAE results under different parameters.

**Figure 11:**
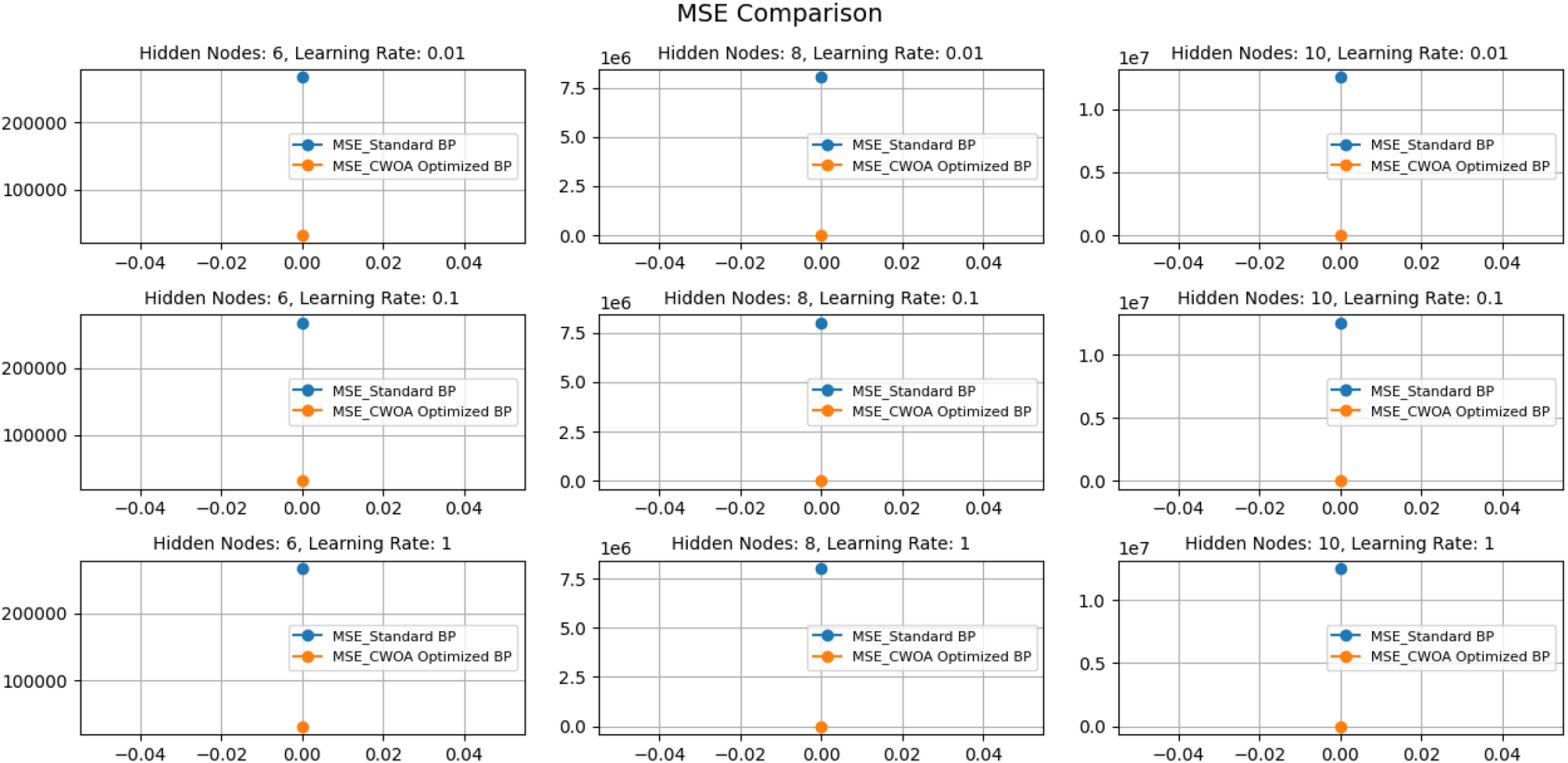
Comparison of MSE results under different parameters.

Based on the results of Figs. 10 and 11, the following conclusions can be drawn:

1. The learning rate significantly impacts the model performance. Under the number of hidden nodes, the Mean absolute error (MAE) at the learning rate 1 is significantly lower than other learning rates. This indicates that a higher learning rate on this dataset can help the model make predictions more accurately.
2. The number of hidden nodes also impacts model performance: at all learning rates, the MAE is the lowest when the number of hidden nodes is 10, while the MAE is higher when the number of hidden nodes is 6 and 8. This indicates that increasing the number of hidden nodes can help improve the model's predictive performance, especially in more complex problems.
3. The CWOA-optimized BP neural network performs better than the standard BP neural network. Regardless of the learning rate and the number of hidden nodes, the MAE of the CWOA-optimized BP neural network is significantly lower than the standard BP neural network. This indicates that using the CWOA optimization algorithm can improve the training performance of the model and reduce prediction errors.

Based on this result, we can infer that a higher learning rate and an appropriate number of hidden nodes can improve the predictive performance of the BP neural network on this dataset. In addition, using the CWOA optimization algorithm can further improve the training effect of the model and reduce prediction errors. However, we still need to conduct more experiments and validation to draw more reliable conclusions.

**Figure 12:**
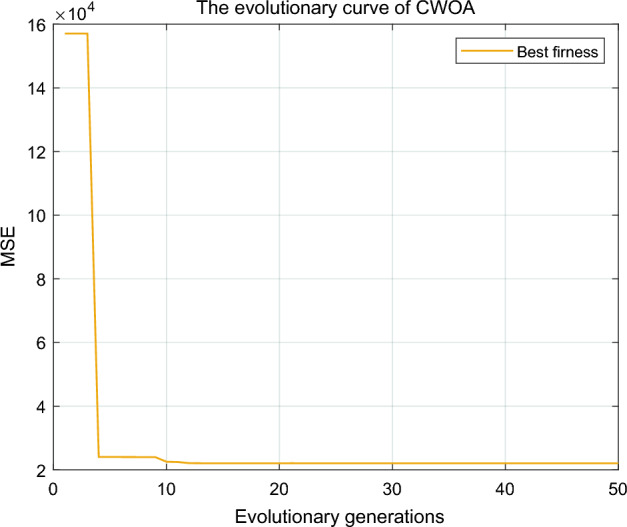
Convergence curve of CWOA.

The Lasso method selects the three variables with the most significant impact on GDP as time series inputs. The macro code is written in Excel, and the training set samples are generated using the sliding window method as input to the neural network's input layer. Based on the above analysis, the optimal parameters were selected, and Fig. 12 shows the convergence curve of CWOA during the optimization process.

After the above tests, using the constructed model to forecast and analyze the test set data for 2019–2021, the GDP forecast table is obtained as follows Tables 5 and 6:

**Table 5:** 2019–2021 GDP forecast value (Unit: 100 million yuan).

Year	Actual value	Predicted value	Error	Relative error (%)
2019	23,605.77	23,030	− 575.77	2.43
2020	25,002.79	25,430	427.21	1.70
2021	27,894.02	27,590	− 304.02	1.08

**Table 6:** 2019–2021 GDP forecast under 80% and 95% confidence intervals (Unit: 100 million yuan).

Year	Actual value	Predicted value	80%Confidence interval	95%Confidence interval
2019	23,605.77	23,030	(21,616.86, 24,722.03)	(20,862.38,25,615.94)
2020	25,002.79	25,430	(21,292.94, 28,524.82)	(19,707.19, 30,820.03)
2021	27,894.02	27,590	(20,610.44, 33,217.11)	(18,164.58, 37,689.62)

**Figure 13:**
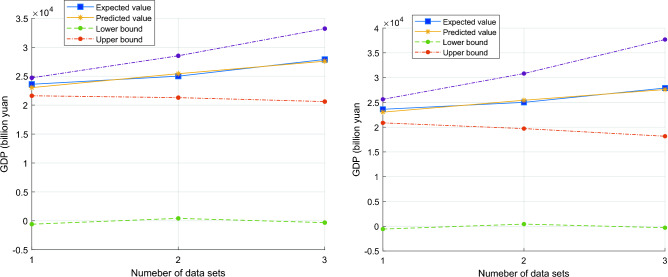
Comparison of measured and predicted values at 80% and 95% confidence level.

By establishing the Lasso–CWOA–BP model to predict Chongqing's GDP, the relative error is found to be within 5%. Upon calculation, the average absolute percentage error (MAPE = 0.017484), root mean square error (RMSE = 450.1514), average absolute error (MAE = 436.3841), and determination coefficient of the economic data sequence under the predicted results are obtained, which is 0.950. These results suggest the model can be used as a reliable Chongqing GDP forecast model. Figure 13 compares measured and predicted values at 80% and 95% confidence levels.

#### Empirical analysis of combination model

In order to analyze and predict the GDP of Chongqing from 2019 to 2021, both the ARIMA model and the improved CWOA–BP neural network model were utilized. The ARIMA time series model showed an accuracy of 97.6% compared to actual data, while the improved COWA–BP neural network prediction model achieved a higher accuracy of 98.2%. Although the accuracy of these single prediction models is high, this paper aims to achieve optimal prediction performance through a combined prediction model that utilizes the error variance mean square reciprocal method.

The combination forecasting model is used to predict the GDP of Chongqing, and the relative error is within 5%. After calculation, the mean absolute percentage error (MAPE = 0.012), root mean square error (RMSE = 357.603), mean absolute error (MAE = 301.904), and determination coefficient are 0.970, which can be used as the GDP prediction model of Chongqing.

### Multi-model comparative analysis

To further accentuate the strengths of our proposed model, this paper conducts a comprehensive comparison with a diverse set of models. Alongside our proposed model, this paper includes well-established techniques such as Random Forest, Gradient Boosting, K-Nearest Neighbors, Decision Tree, Multi-layer Perceptron, and XGBoost. Additionally, we introduce novel approaches like GA–BP and WOA–BP to enrich the multi-model analysis. Studies similar to this data set in the past 5 years were selected as the comparison model, as shown in the following Table 7:

**Table 7:** Some similar studies in this field in recent 5 year.

Year	Method used	Reference
2022	Random forest boosting algorithms	Chu and Qureshi^32^
2021	K-nearest neighborhood ARIMA	Maccarrone et al.^33^
2021	Gradient boosting random forest	Yoon^34^
2021	Decision tree random forest	Soybilgen and Yazgan^35^
2019	Multi-layer perceptron	Gaffar and Gaffar^36^
2020	Boosting random forest	Maehashi and Shintani^37^
2023	GA–BP neural network	Yu^38^
2023	XGBoost	Chen et al.^39^

**Figure 14:**
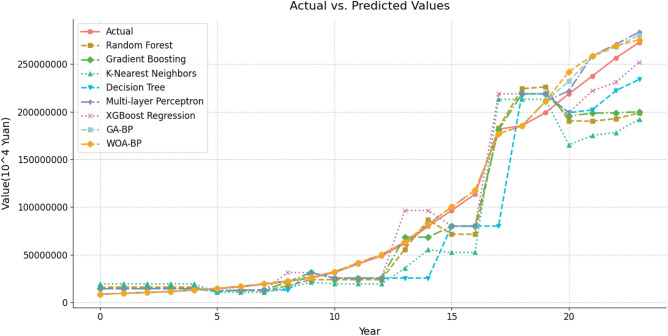
A comparison of the actual data with each prediction model.

After conducting experiments and evaluating the performance of these classic learning techniques in predicting GDP data, this paper presents the results in a comprehensive visualization. The Fig. 14 displays the actual GDP values of Chongqing over the years alongside the predicted values obtained through each method. This visualization provides a clear depiction of how well each technology captures the actual GDP trends during this period.

Having thoroughly assessed the performance and capabilities of various predictive models in GDP prediction, we now present a comprehensive comparison table summarizing the key evaluation metrics for each model. The table titled 'Comparison Table of GDP Prediction Model Performance' (Table 8) provides a concise overview of the Mean Absolute Error (MAE), Root Mean Squared Error (RMSE), and Relative Error achieved by each model. Through this tabular representation, readers can easily grasp the relative strengths and weaknesses of each method in terms of predictive accuracy and generalization capability. This table serves as a valuable reference for understanding the model's performance and making informed decisions when applying these techniques in real-world GDP forecasting scenarios.

**Table 8:** Comparison table of GDP prediction model performance.

Model	MAE (10^4^)	RMSE (10^4^)	Relative error (%)
Random forest	6165.26	6262.40	23.96
Gradient boosting	5638.39	5809.66	21.82
K-Nearest neighbors	7362.86	7407.65	28.73
Decision tree	3618.81	3623.43	14.18
Multi-layer perceptron	1556.65	1616.64	6.21
XGBoost	2052.15	2092.41	8.01
GA–BP	1331.16	1430.48	5.34
WOA–BP	1231.16	1444.21	5.02
ARIMA	624.95	689.14	2.40
CWOA–BP	435.67	449.61	1.75
CWOA–BP–ARIMA	301.90	357.60	1.20

**Figure 15:**
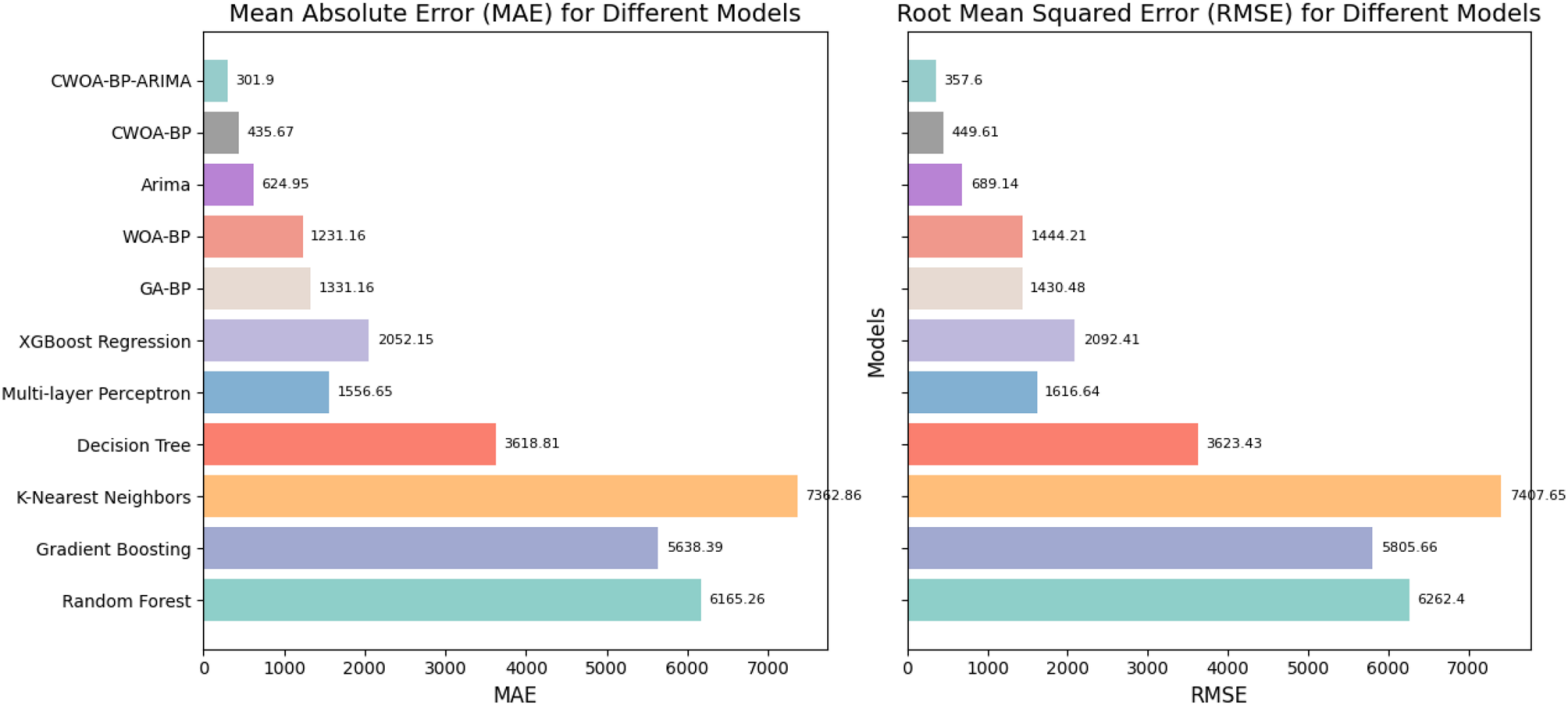
Model comparison: performance metrics.

**Figure16:**
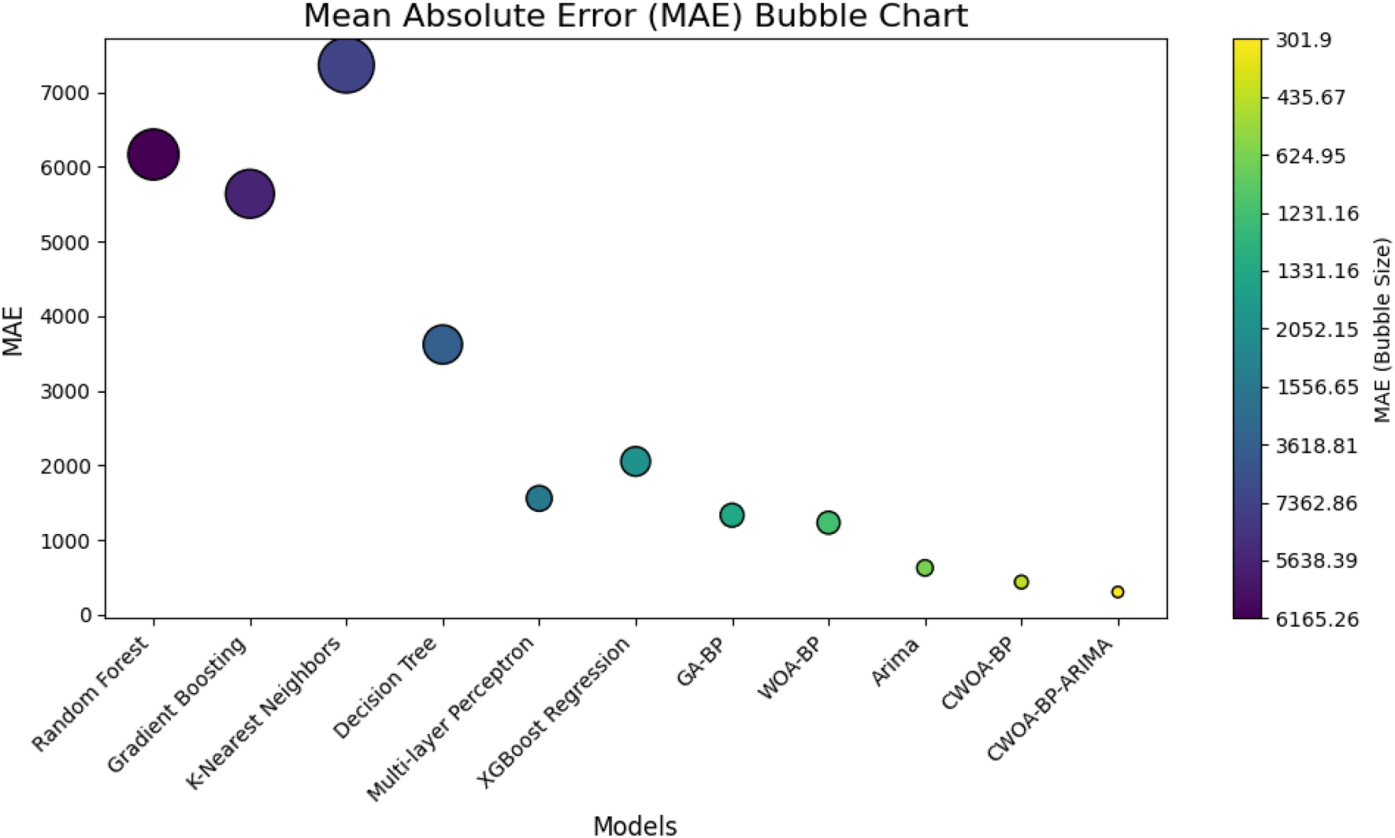
MAE bubble diagrams for different models.

In Figs. 15 and 16**,** we present comprehensive visualizations to assess the performance of various predictive models in GDP prediction. Figure 15 showcases the MAE and RMSE for each model, providing valuable insights into their accuracy and deviation capturing abilities. In contrast, Fig. 16 introduces an innovative MAE Bubble Chart, offering a dynamic representation of the models' comparative accuracy through bubble sizes. These visuals enable informed decision-making, guiding the selection of the most suitable model for precise GDP forecasting in real-world scenarios.

The CWOA–BP–ARIMA model exhibits significant advantages over Random Forest, MLP, GA–BP, and CWOA–BP models in GDP prediction. It achieves a remarkable MAE reduction of 95% and RMSE reduction of 94.2% compared to Random Forest. When compared to MLP, the model shows a substantial MAE reduction of 80.6% and RMSE reduction of 77.8%. Additionally, relative to GA–BP, the model demonstrates a considerable MAE reduction of 77.3% and RMSE reduction of 75%. Moreover, in comparison to its own CWOA–BP counterpart, the model achieves an impressive MAE reduction of 30.7% and RMSE reduction of 20.46%. These results highlight the superior predictive accuracy and robustness of the CWOA–BP–ARIMA model, making it a reliable forecasting tool for practical economic planning and decision-making.

In conclusion, the pursuit of advanced predictive models in GDP forecasting holds immense significance. Our findings act as a guiding compass, leading researchers and practitioners towards precise economic predictions. With CWOA–BP and CWOA–BP–ARIMA shining as beacons of accuracy and resilience, this paper unlocks unparalleled efficiency and trustworthiness in real-world applications. These groundbreaking advancements enrich the landscape of economic forecasting, paving the way for further innovation and enhanced predictive capabilities.

### Forecasting the next 3 years based on the optimal model

**Figure 17:**
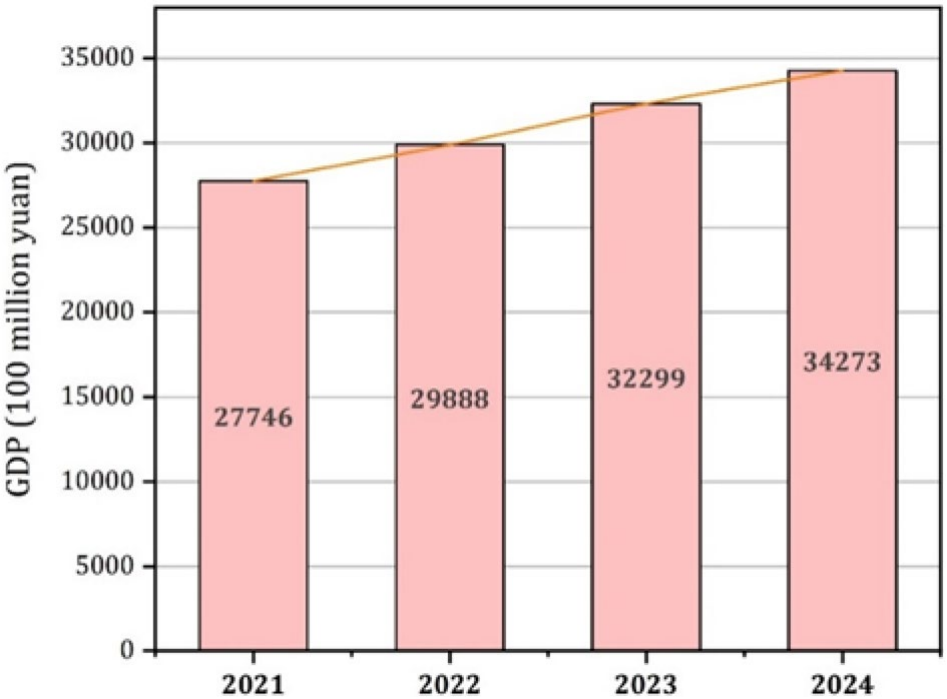
The GDP forecast of Chongqing in the next 3 years.

The improved combination forecasting model is used to predict the GDP of Chongqing from 2022 to 2024, and the prediction results are shown in Fig. 17 and Table 9. The GDP of Chongqing has been steadily rising over time. By scientifically analyzing historical GDP data, social workers can better understand the national economy and its development trends and changes. This paper uses Chongqing's GDP as the research object, develops various models, and compares them to determine the best model for predicting GDP based on these two points. The study's findings, based on Chongqing, can be used as a model for economic growth and as a reasonable basis for the departments in question to develop economic policies.

**Table 9:** 2022–2024 GDP forecast under 80% and 95% confidence intervals (Unit: 100 million yuan).

Year	Predicted value	80% Confidence interval	95% Confidence interval
2022	29,888	(23,057.17, 36,688.55)	(20,389.88, 41,488.02)
2023	32,299	(21,811.25, 42,357.47)	(18,297.23, 50,492.51)
2024	34,273	(20,423.84,49,123.36)	(16,190.23, 61,968.41)

## Conclusions and discussions

This study presents a novel point interval GDP prediction model based on the Lasso method, dedicated to enhancing the accuracy of short-term GDP forecasts. Through extensive experimentation and rigorous evaluation, the model has demonstrated its superiority over several benchmark models in terms of accuracy and predictive capability.

Among the evaluated models, CWOA–BP and CWOA–BP–ARIMA emerge as standout performers, showcasing exceptional accuracy and robustness with the lowest MAE, RMSE, and Relative Error values compared to others. These models hold great promise for practical GDP forecasting applications, offering reliable insights for decision-making processes.

The incorporation of the Lasso method in conjunction with the point interval approach empowers the model to capture and quantify the uncertainty surrounding GDP predictions. This comprehensive understanding of forecast uncertainty equips policymakers and analysts with valuable information to navigate potential economic fluctuations with confidence.

In summary, this research contributes significantly to the field of GDP forecasting by presenting a highly effective predictive model. By leveraging the strengths of CWOA–BP and CWOA–BP–ARIMA, this innovative approach unlocks unprecedented efficiency and trustworthiness in real-world applications. As we continue to advance predictive modeling techniques, the implications of this study provide valuable guidance for researchers and practitioners seeking to optimize GDP predictions for economic planning and policymaking.

## Data Availability

The data used to support the findings of this study is available from the corresponding author upon request.
